# Supplementary material for: Public transit mobility as a leading indicator of COVID-19 transmission in 40 cities during the first wave of the pandemic
Source: PeerJ. 2024 May 31;12:e17455. doi: 10.7717/peerj.17455 (PMC11146320; doi:10.7717/peerj.17455)
Supplement: Supplemental Information 1 [file peerj-12-17455-s001.docx]

**Table S1.** Date when the first major physical distancing intervention (restrictions on public gatherings or mandatory business closures) was announced by national or sub-national government.

| **Country** | **City** | **Geography of restriction** | **Date announced** | **Restriction type** |
| --- | --- | --- | --- | --- |
| **Australia** | | | | |
| **Australia** | Melbourne | National | March 13 [1] | Ban gatherings > 500 people |
|  | Sydney |  |  |  |
| **Europe** | | | | |
| **Austria** | Vienna | National | March 10 [2] | Ban gatherings > 500 outdoors  Ban gatherings > 100 indoors |
| **Belgium** | Brussels | National | March 10 [3] | Ban gatherings > 1,000 people indoors |
| **Denmark** | Copenhagen | National | March 06 [4] | Ban gatherings > 1,000 people |
| **France** | Lyon | National | March 09 [5] | Ban gatherings > 1,000 people |
|  | Paris |  |  |  |
| **Germany** | Berlin | National | March 08 [6] | Ban gatherings > 1,000 people |
|  | Hamburg |  |  |  |
|  | Rhine-Ruhr |  |  |  |
| **Italy** | Milan | Sub-national (Lombardia) | March 08 [7] | Quarantine – Northern Italy |
|  | Rome | National | March 09 [8] | Quarantine – Nationwide |
| **Netherlands** | Amsterdam | National | March 12 [10] | Ban gatherings > 100 people |
| **Portugal** | Lisbon | National | March 14 [11] | Ban gatherings > 100 people |
| **Russia** | Moscow | National | March 24 [12] | Emergency measures (closures of nightclubs, cinemas, etc.) |
|  | St. Petersburg |  |  |  |
| **Spain** | Barcelona | National | March 13 [13] | State of emergency, restrictions on free movement |
|  | Madrid |  |  |  |
| **Sweden** | Stockholm | National | March 11 [14] | Ban gatherings > 500 people |
| **Turkey** | Istanbul | National | March 12 [15] | Closure of “places where people tend to mingle” |
| **United Kingdom** | Birmingham | National | March 16 [16] | Advised to avoid gatherings and crowded places, work from home where possible |
|  | London |  |  |  |
|  | Manchester |  |  |  |
| **Canada and the United States** | | | | |
| **Canada** | Vancouver | Sub-national (British Columbia) | March 12 [17] | Ban gatherings >250 people |
|  | Montreal | Sub-national (Quebec) | March 13 [18] | Ban gatherings >250 people |
|  | Toronto | Sub-national (Ontario) | March 13 [19] | Ban gatherings >250 people |
| **United States** | Los Angeles | Sub-national (California) | March 11^a^ [20] | Bans gatherings >250 people |
|  | San Francisco |  |  |  |
|  | Seattle | Sub-national (Washington) | March 11^a^ [21] | Bans gatherings >250 people |
|  | New York City | Sub-national (New York) | March 12^a^ [22] | Bans gatherings >500 people |
|  | Philadelphia | Sub-national (Pennsylvania) | March 12^a^ [23] | Bans gatherings >250 people |
|  | Boston | Sub-national (Massachusetts) | March 13^a^ [24] | Bans gatherings >250 people |
|  | Chicago | Sub-national (Illinois) | March 13^a^ [25] | Bans gatherings >1000 people |
|  | Washington DC | Sub-national (District of Columbia) | March 13^a^ [26] | Bans gatherings >250 people |
| **Latin America** | | | | |
| **Mexico** | Mexico City | National | March 14 [27] | Cancellation of sporting and events in schools; school closures |
| **Brazil** | São Paulo | Sub-national (São Paulo) | March 13 [28] | Bans gatherings >500 people |
| **Asia** | | | | |
| **Hong Kong** | Hong Kong | National | January 29 [29] | Closure of public facilities |
| **Japan** | Tokyo | National | February 27 [30] | School closures, people urged to avoid congregating in enclosed spaces |
| **South Korea** | Seoul | Local | February 21 [31] | Closure of public spaces, protests banned |
| **Singapore** | Singapore | National | March 20 [32] | Ban gatherings >250 people |
| ^a^ Regional state of emergency declared before this date  [1] Australia: <https://www.abc.net.au/news/2020-03-13/coronavirus-scott-morrison-coag-premiers-cancelling-events/12053382>  [2] Austria: <https://orf.at/stories/3157262/>  [3] Belgium: <https://www.brusselstimes.com/belgium/99636/brussels-cancels-concerts-with-over-1000-people-from-wednesday-covid-19>  [4] Denmark: <https://www.dr.dk/nyheder/indland/undgaa-visse-rejser-og-drop-tog-i-myldretiden-her-er-myndighedernes-corona>  [5] France: <https://www.rfi.fr/en/france/20200309-france-bans-public-gatherings-1000-people-over-coronavirus>  [6] Germany: [https://web.archive.org/web/20200320150813/https://www.mt.de/nrw/22720700_Landesregierung-Grossveranstaltungen-in-NRW-wegen-Corona-Virus-grundsaetzlich-absagen.html](https://web.archive.org/web/20200320150813/https:/www.mt.de/nrw/22720700_Landesregierung-Grossveranstaltungen-in-NRW-wegen-Corona-Virus-grundsaetzlich-absagen.html)  [7] Italy – Milan: <https://www.bbc.com/news/world-middle-east-51787238>  [8] Italy – Nationwide: <https://www.bbc.com/news/world-europe-51810673>  [9] Monaco: <https://forbes.mc/article/coronavirus-monaco-bans-gatherings-of-more-than-100-people>  [10] Netherlands: <https://nationalpost.com/pmn/health-pmn/dutch-ban-big-public-events-over-coronavirus>  [11] Portugal: <https://www.bloomberg.com/news/articles/2020-03-14/widespread-clampdown-takes-hold-spanish-emergency-virus-update-k7s4ru6n>  [12] Russia: <http://government.ru/orders/selection/401/39260/>  [13] Spain: <https://www.pbs.org/newshour/health/spain-to-declare-state-of-emergency-over-virus-outbreak>  [14] Sweden: <https://www.bloomberg.com/news/articles/2020-03-11/sweden-health-agency-seeks-ban-on-meetings-of-500-people-or-more>  [15] Turkey: <https://www.bloomberg.com/news/articles/2020-03-12/turkey-screens-europe-bound-refugees-for-coronavirus>  [16] United Kingdom: <https://www.bbc.com/news/uk-51917562>  [17] Canada – British Columbia: <https://www.vicnews.com/news/covid-19-province-bans-large-gatherings-in-b-c/>  [18] Canada – Quebec: <https://montrealgazette.com/news/local-news/covid-19-montreal-st-patricks-parade-postponed-events-drawing-250-or-more-cancelled/>  [19] Canada – Ontario: <https://globalnews.ca/news/6672452/ontario-health-official-coronavirus-events/>  [20] USA – California: <https://www.gov.ca.gov/2020/03/11/california-public-health-experts-mass-gatherings-should-be-postponed-or-canceled-statewide-to-slow-the-spread-of-covid-19/>  [21] USA – Washington State: <https://coronavirus.dc.gov/sites/default/files/dc/sites/coronavirus/release_content/attachments/DOH_Rulemaking_Mass-Gatherings.pdf>  [22] USA – New York: <https://www.governor.ny.gov/news/during-novel-coronavirus-briefing-governor-cuomo-announces-new-mass-gatherings-regulations>  [23] USA – Philadelphia: <https://www.timesleader.com/news/776061/wolf-recommends-suspending-large-gatherings-discourages-non-essential-travel>  [24] USA – Massachusetts: <https://www.mass.gov/doc/order-prohibiting-gatherings-of-more-than-250-people/download>  [25] USA – Illinois: <https://www2.illinois.gov/Pages/Executive-Orders/ExecutiveOrder2020-04.aspx>  [26] USA – Washington: [https://www.governor.wa.gov/sites/default/files/20-07%20Coronavirus%20%28tmp%29.pdf](https://www.governor.wa.gov/sites/default/files/20-07%20Coronavirus%20(tmp).pdf)  [27] Mexico: <https://www.eluniversal.com.mx/nacion/coronavirus-sep-cancela-eventos-deportivos-y-civicos-en-escuelas>; <https://www.am.com.mx/noticias/Vacaciones-de-Semana-Santa-se-adelantan-y-extienden-por-coronavirus-20200314-0021.html>  [28] Brazil – São Paulo: <https://g1.globo.com/sp/sao-paulo/noticia/2020/03/13/prefeitura-de-sao-paulo-cancela-todos-os-eventos-de-massa-apos-casos-de-coronavirus.ghtml>  [29] Hong Kong: <https://www.lcsd.gov.hk/clpss/en/webApp/NewsDetails.do?id=14683>; <https://fortune.com/2020/03/19/coronavirus-hong-kong-quarantine-tracking/>  [30] Japan: <https://www.vox.com/covid-19-coronavirus-explainers/2020/3/28/21196382/japan-coronavirus-cases-covid-19-deaths-quarantine>; <https://business.financialpost.com/pmn/business-pmn/japan-to-create-fund-to-subsidize-parents-during-school-closure-nikkei>  [31] South Korea – Seoul: <https://www.nytimes.com/2020/02/23/world/asia/china-coronavirus.html>  [32] Singapore: <https://www.channelnewsasia.com/news/singapore/covid19-events-gatherings-suspended-june-250-participants-12560620> | | | | |
