# Supplementary material for: Public transit mobility as a leading indicator of COVID-19 transmission in 40 cities during the first wave of the pandemic
Source: PeerJ. 2024 May 31;12:e17455. doi: 10.7717/peerj.17455 (PMC11146320; doi:10.7717/peerj.17455)
Supplement: Supplemental Information 2 [file peerj-12-17455-s002.docx]

**Table S2.** Sources and level of geography for COVID-19 case data corresponding to each city in the dataset.

| **Country** | **City** | **Geography of case data** | **Source** |
| --- | --- | --- | --- |
| **Australia** | | | |
| **Australia** | Melbourne | Local Government Areas (LGAs) within the Primary Health Networks (PHNs) of Eastern Melbourne, North Western Melbourne, and South Eastern Melbourne | Victoria State Government [1] |
|  | Sydney | Local Health Districts (LHDs) of Northern Sydney, South Eastern Sydney, South Western Sydney, Sydney, and Western Sydney | Government of New South Whales [2] |
| **Europe** | | | |
| **Austria** | Vienna | City (Vienna) | Google [3] |
| **Belgium** | Brussels | City (Brussels) | Google [3] |
| **Denmark** | Copenhagen | National (Denmark) | Google [3] |
| **France** | Lyon | Department (Rhône) | Google [3] |
|  | Paris | City (Paris) |  |
| **Germany** | Berlin | City (Berlin) | Google [3] |
|  | Hamburg | City (Hamburg) |  |
|  | Rhine-Ruhr | Regions of Dortmund, Essen, Duisburg, Bochum, Gelsenkirchen, Oberhausen, Düsseldorf, Rhein-Kreis Neuss, Mönchengladbach, Wuppertal, Cologne, Bonn, Leverkusen |  |
| **Italy** | Milan | City (Milano) | Google [3] |
|  | Rome | City (Roma) |  |
| **Netherlands** | Amsterdam | City (Amsterdam) | Google [3] |
| **Portugal** | Lisbon | Region (Lisbon Metropolitan Area) | Google [3] |
| **Russia** | Moscow | City (Moscow) | Yandex [4] |
|  | St. Petersburg | City (St. Petersburg) | Yandex [5] |
| **Spain** | Barcelona | City (Barcelona) | Google [3] |
|  | Madrid | City (Madrid) |  |
| **Sweden** | Stockholm | County (Stockholm) | Google [3] |
| **Turkey** | Istanbul | National (Turkey) | Google [3] |
| **United Kingdom** | Birmingham | City (Birmingham) | Google [3] |
|  | London | Region (London) |  |
|  | Manchester | City (Manchester) |  |
| **Canada and the United States** | | | |
| **Canada** | Montreal | Health region (Montreal) | COVID-19 Canada Open Data Working Group [6] |
|  | Toronto | Health region (Toronto) |  |
|  | Vancouver | Health region (Vancouver Coastal) |  |
| **United States** | Boston | County (Suffolk County) | Google [3] |
|  | Chicago | County (Cook County) |  |
|  | Los Angeles | County (Los Angeles County) |  |
|  | New York City | City (New York City) |  |
|  | Philadelphia | County (Philadelphia County) |  |
|  | San Francisco | City (San Francisco) |  |
|  | Seattle | County (King County) |  |
|  | Washington DC | City (Washington DC) |  |
| **Latin America** | | | |
| **Mexico** | Mexico City | National (Mexico) | Google [3] |
| **Brazil** | São Paulo | City (São Paulo) | Wesley Cota [7] |
| **Asia** | | | |
| **Hong Kong** | Hong Kong | National (Hong Kong) | Google [3] |
| **Japan** | Tokyo | City (Tokyo) | Tokyo Metropolitan Government [8] |
| **South Korea** | Seoul | City (Seoul) | Google [3] |
| **Singapore** | Singapore | National (Singapore) | Google [3] |
| [1] Victoria State Government: <https://www.coronavirus.vic.gov.au/victorian-coronavirus-covid-19-data>  [2] Government of New South Whales: <https://data.nsw.gov.au/search/dataset/ds-nsw-ckan-aefcde60-3b0c-4bc0-9af1-6fe652944ec2/details>  [3] Google: <https://health.google.com/covid-19/open-data/>  [4] Yandex: <https://yandex.ru/web-maps/covid19?ll=37.646921%2C55.725146&z=9>  [5] Yandex: <https://yandex.ru/web-maps/covid19?ll=30.424830%2C59.939314&z=9>  [6] COVID-19 Canada Open Data Working Group: <https://github.com/ccodwg/CovidTimelineCanada>  [7] Wesley Cota: <https://github.com/wcota/covid19br/>  [8] Tokyo Metropolitan Government: <https://catalog.data.metro.tokyo.lg.jp/dataset/t000010d0000000068> | | | |
