# Supplementary material for: Public transit mobility as a leading indicator of COVID-19 transmission in 40 cities during the first wave of the pandemic
Source: PeerJ. 2024 May 31;12:e17455. doi: 10.7717/peerj.17455 (PMC11146320; doi:10.7717/peerj.17455)
Supplement: Supplemental Information 3 — Model coefficients are presented with and without adjustment for days since 100th case. Models include outcome data from March 30–April 12 for 40 cities. a Coefficient for a 10% decrease in the mobility index. b Coefficient for a 10-day increase since the 100th reported case in the country. [file peerj-12-17455-s003.docx]

**Table S3.** Estimated model coefficients (with 95% confidence intervals) for the association between a 10% decrease in the mobility index and the weekly case ratio and effective reproduction number assuming a lag of two or three weeks and using two weeks of data.

Model coefficients are presented with and without adjustment for days since 100^th^ case. Models include outcome data from March 30–April 12 for 40 cities.

|  | **Weekly case ratio (exp(*β*) = ratio of current week to previous week)** | | | |
| --- | --- | --- | --- | --- |
|  | **2-week lag** | | **3-week lag** | |
|  | **Unadjusted** | **Adjusted** | **Unadjusted** | **Adjusted** |
| Mobility^a^ | 0.848 (0.787, 0.913) | 0.898 (0.829, 0.973) | 0.928 (0.902, 0.956) | 0.948 (0.906, 0.991) |
| Days since 100^th^ case^b^ |  | 0.787 (0.658, 0.941) |  | 0.867 (0.683, 1.101) |
| Marginal *R^2^* | 0.238 | 0.291 | 0.164 | 0.200 |
| Conditional *R^2^* | 0.690 | 0.714 | 0.636 | 0.659 |
| Likelihood ratio test | *χ^2^*(1) = 6.728, *p* = 0.009 | | *χ^2^*(1) = 1.369, *p* = 0.242 | |
|  | **Effective reproduction number (*β* = *ΔR_t_*)** | | | |
|  | **2-week lag** | | **3-week lag** | |
|  | **Unadjusted** | **Adjusted** | **Unadjusted** | **Adjusted** |
| Mobility^a^ | -0.068 (-0.100, -0.036) | -0.041 (-0.077, -0.005) | -0.034 (-0.046, -0.022) | -0.026 (-0.046, -0.007) |
| Days since 100^th^ case^b^ |  | -0.113 (-0.195, -0.031) |  | -0.049 (-0.155, 0.057) |
| Marginal *R^2^* | 0.211 | 0.268 | 0.167 | 0.193 |
| Conditional *R^2^* | 0.680 | 0.730 | 0.690 | 0.705 |
| Likelihood ratio test | *χ^2^*(1) = 7.349, *p* = 0.007 | | *χ^2^*(1) = 0.835, *p* = 0.361 | |

^a^ Coefficient for a 10% decrease in the mobility index.
^b^ Coefficient for a 10-day increase since the 100^th^ reported case in the country.
