# Supplementary figures and images for: Public transit mobility as a leading indicator of COVID-19 transmission in 40 cities during the first wave of the pandemic
Source: PeerJ. 2024 May 31;12:e17455. doi: 10.7717/peerj.17455 (PMC11146320; doi:10.7717/peerj.17455)

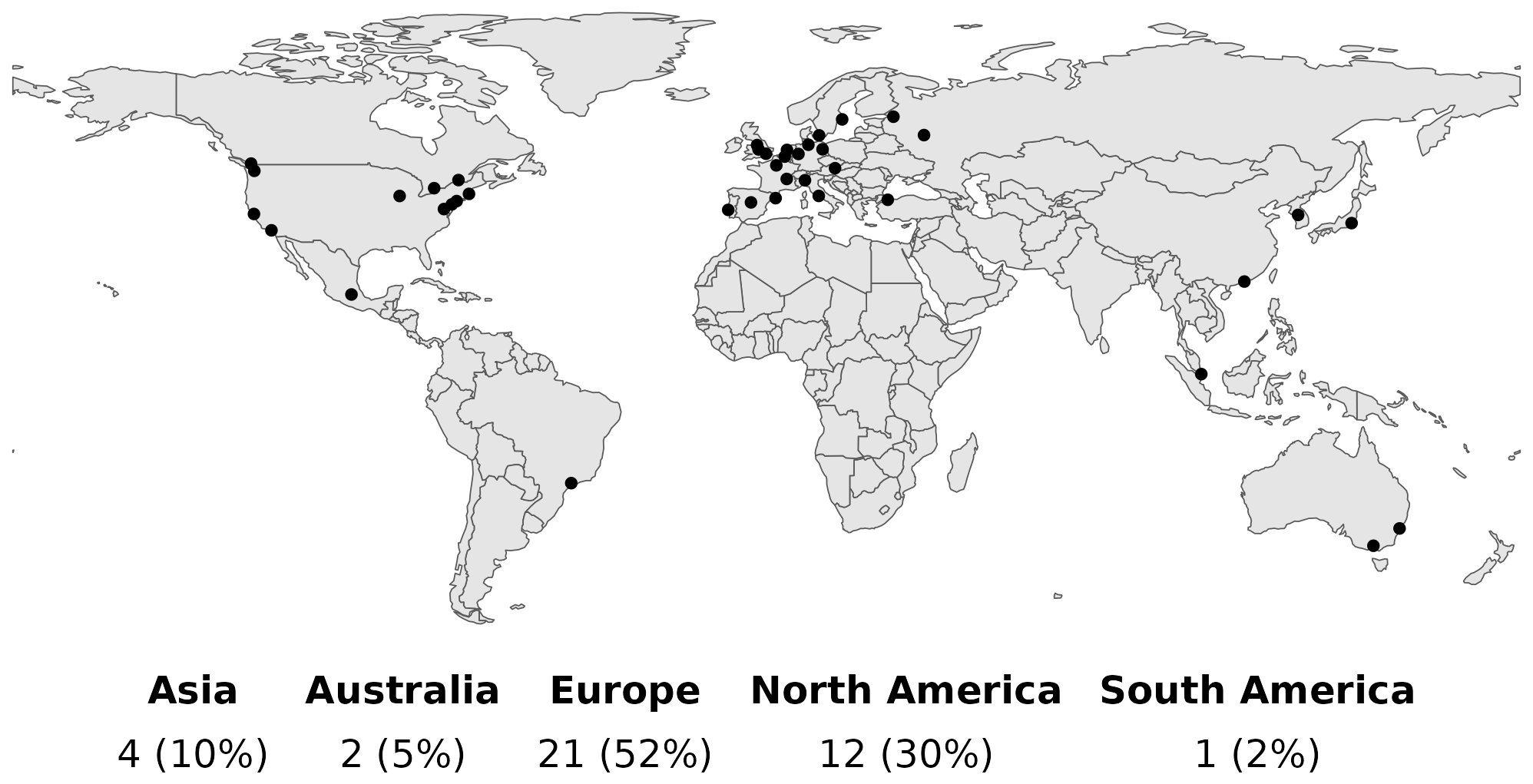

Supplement: Supplemental Information 4 — Made with Natural Earth. [file peerj-12-17455-s004.png]

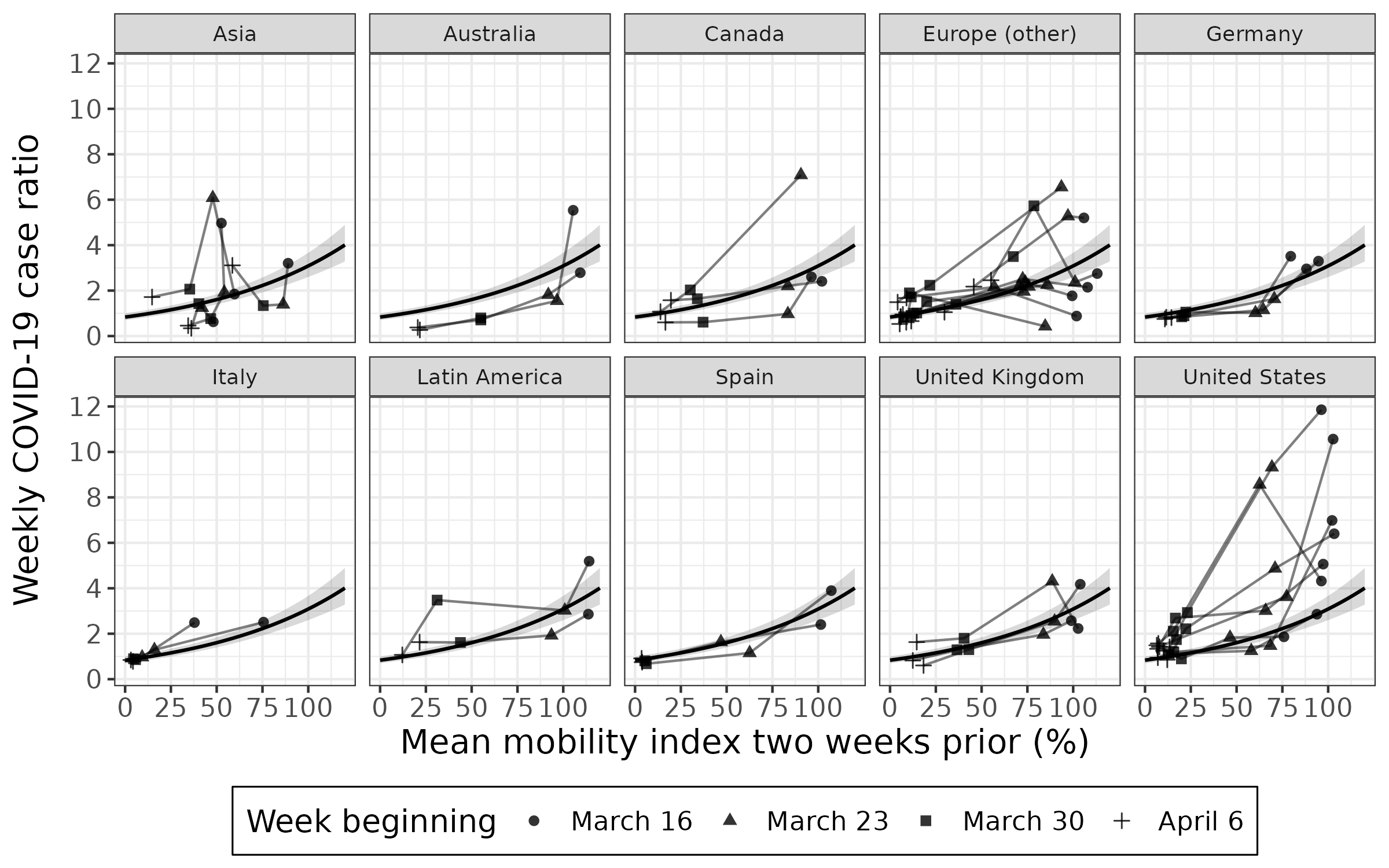

Supplement: Supplemental Information 5 — Each point represents a week of data for a given city. The four-week trajectory for each city is plotted with a line connecting adjoining weeks. A multilevel model was fit on all four weeks of data. The 95% confidence interval of the fitted value is shown in grey. The first week of data (March 16) excludes Amsterdam, Istanbul, Lyon, Montréal, and Paris. AU = Australia; CA = Canada; DE = Germany; ES = Spain; IT = Italy; UK = United Kingdom; US = United States. [file peerj-12-17455-s005.png]

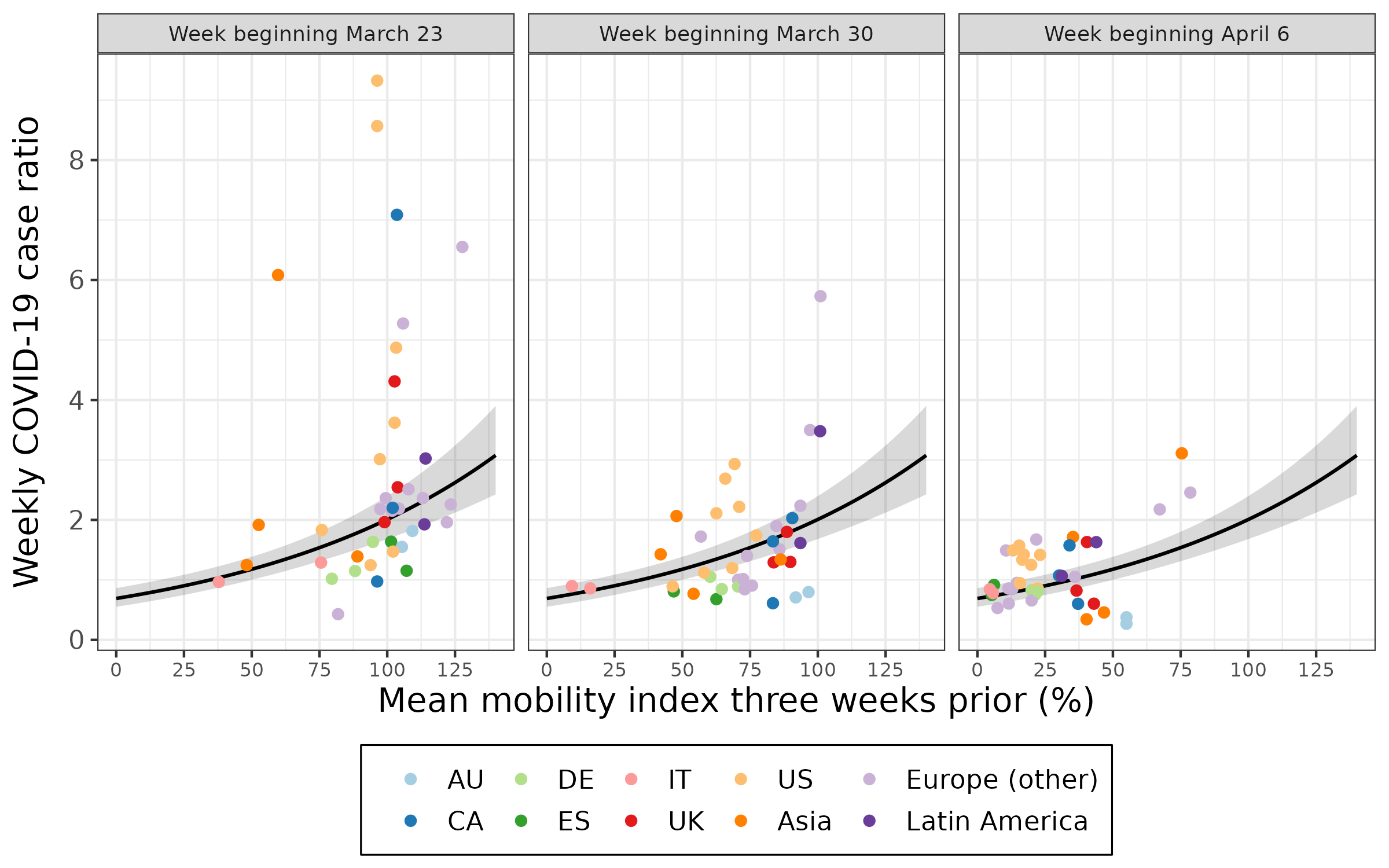

Supplement: Supplemental Information 6 — Each point represents a week of data for a given city. Each week of data (labelled according to the week the outcome was measured) is plotted separately for a multilevel model fit on all three weeks of data. The 95% confidence interval of the fitted value is shown in grey. AU = Australia; CA = Canada; DE = Germany; ES = Spain; IT = Italy; UK = United Kingdom; US = United States. [file peerj-12-17455-s006.png]

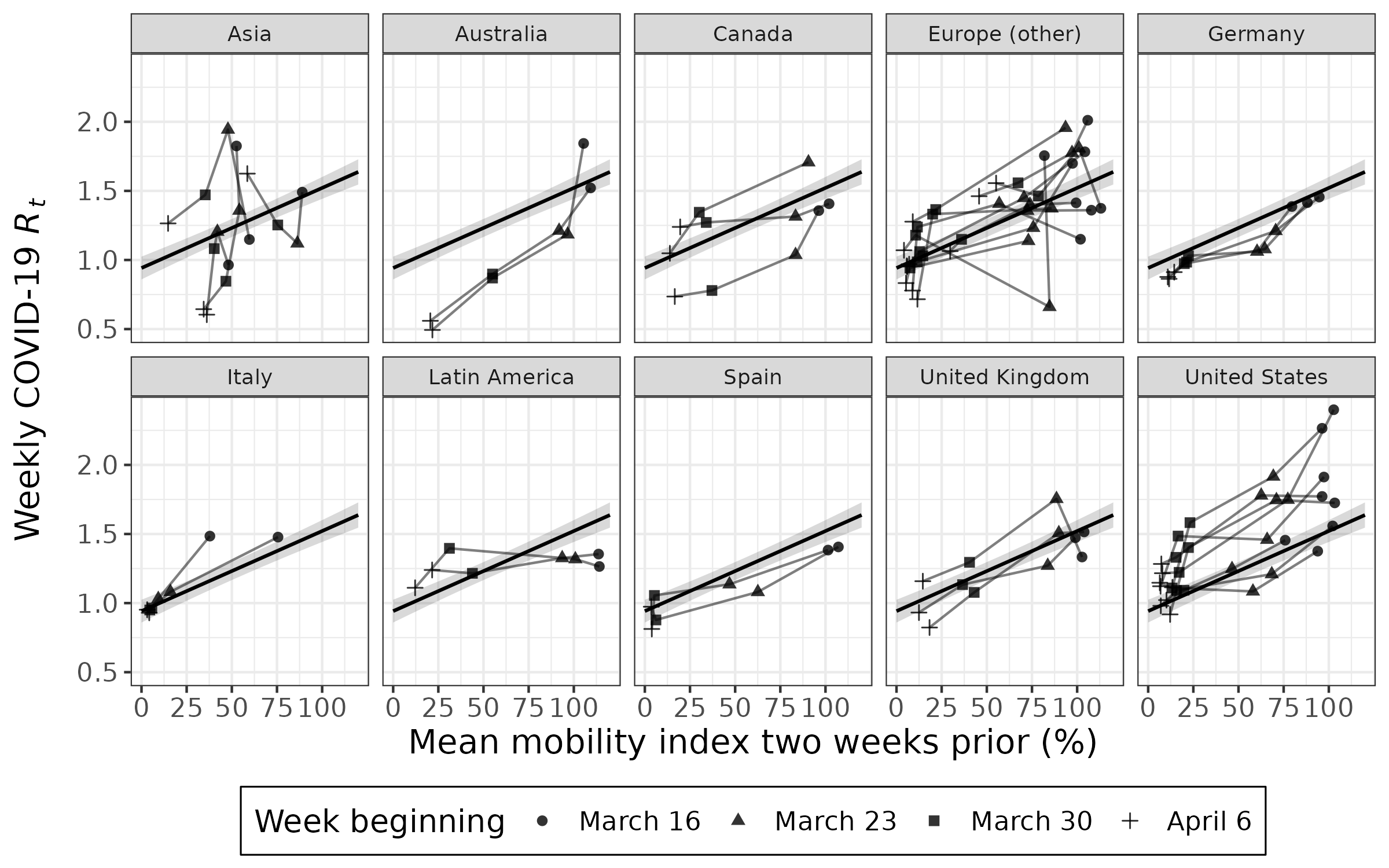

Supplement: Supplemental Information 7 — Each point represents a week of data for a given city. The four-week trajectory for each city is plotted with a line connecting adjoining weeks. A multilevel model was fit on all four weeks of data. The 95% confidence interval of the fitted value is shown in grey. The first week of data (March 16) excludes Istanbul and Montréal. AU = Australia; CA = Canada; DE = Germany; ES = Spain; IT = Italy; UK = United Kingdom; US = United States. [file peerj-12-17455-s007.png]

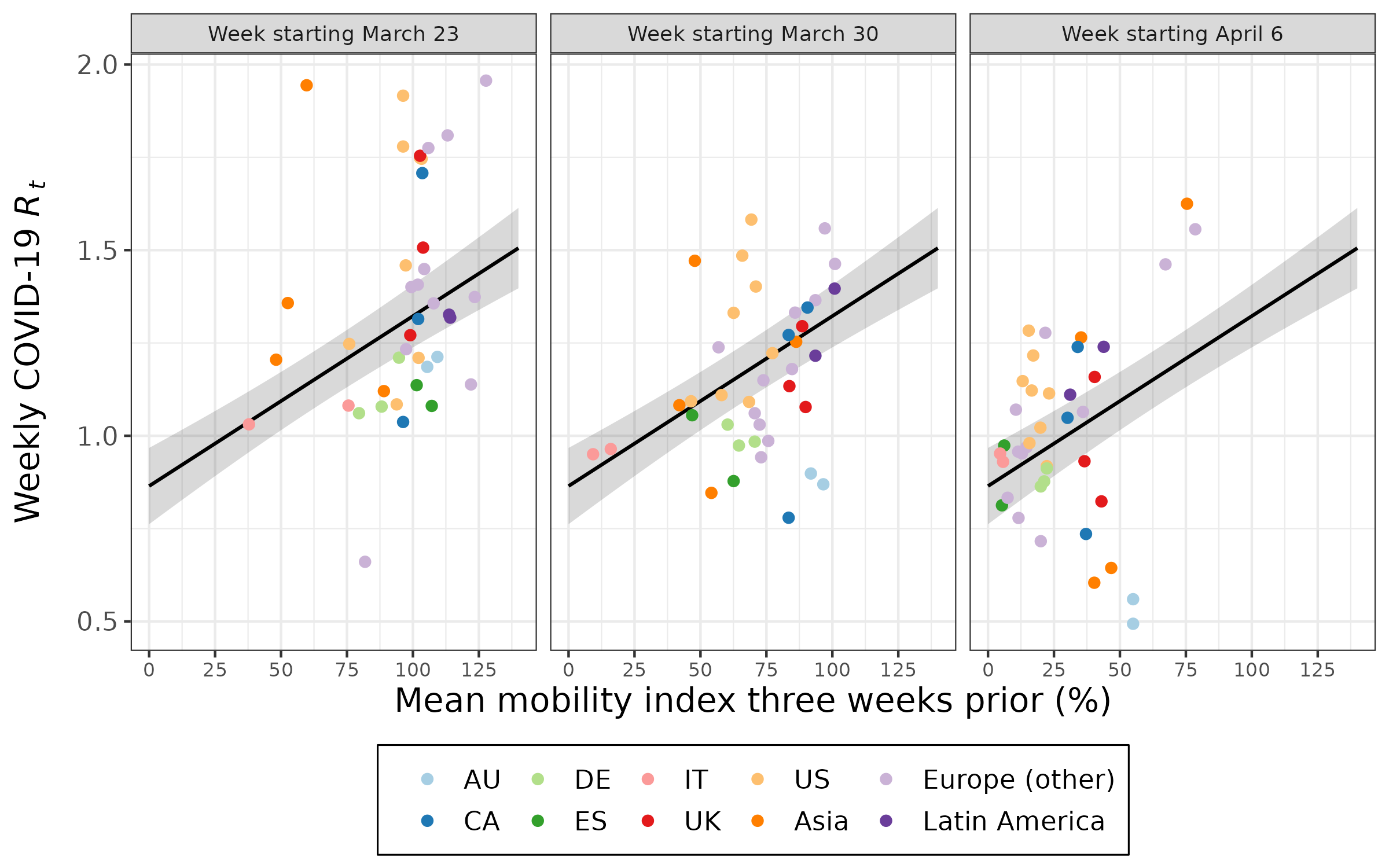

Supplement: Supplemental Information 8 — Each point represents a week of data for a given city. Each week of data (labelled according to the week the outcome was estimated) is plotted separately for a multilevel model fit on all three weeks of data. The 95% confidence interval of the fitted value is shown in grey. AU = Australia; CA = Canada; DE = Germany; ES = Spain; IT = Italy; UK = United Kingdom; US = United States. [file peerj-12-17455-s008.png]
