# Supplementary material for: Public transit mobility as a leading indicator of COVID-19 transmission in 40 cities during the first wave of the pandemic
Source: PeerJ. 2024 May 31;12:e17455. doi: 10.7717/peerj.17455 (PMC11146320; doi:10.7717/peerj.17455)
Supplement: Supplemental Information 9 [file peerj-12-17455-s009.pdf]

Supplementary methods: Public transit mobility as a leading indicator of COVID-19 transmission in 40 cities during the first wave of the pandemic

Jean-Paul R. Soucy, Shelby L. Sturrock, Isha Berry, Duncan J. Westwood, Nick Daneman, David Fisman, Derek R. MacFadden, Kevin A. Brown

For each of our 40 cities, we have up to 4 weeks of outcome data (corresponding to the weeks beginning March 16, March 23, March 30, and April 6, 2020). We denote the outcome for the *i*th observation as y_i, corresponding to a particular week of outcome data from a particular city *k*. We considered models for two different outcomes: the weekly case ratio and the effective reproduction number.

The basic multilevel linear regression model for the weekly case ratio outcome is given as:

$$log(y_i) = \alpha_{jk[i]} + \beta_1 x_{1i} + \epsilon_i$$

In this equation, $log(y_i)$ is the natural logarithm of the *i*th weekly case ratio, $\alpha_{jk[i]}$ is the random intercept for city *k* nested within country *j*, β_1 is the fixed effect of a 1-unit increase in the Citymapper Mobility Index (CMI) two weeks prior (x_{1i}) on $log(y_i)$, and ϵ_i is the error term. The index variables code group membership; for example, *jk[i]* refers to the country *j* and city *k* the *i*th observation belongs to.

In the model adjusted for days since the 100th case in the country, we add an additional term to the regression model:

$$log(y_i) = \alpha_{jk[i]} + \beta_1 x_{1i} + \beta_2 x_{2i} + \epsilon_i$$

In this equation, β_2 is the fixed effect of a 1-unit increase in the number of days since the 100th case in the country (x_{2i}) on $log(y_i)$.

To facilitate interpretation, in the manuscript body and tables, we present β_1 in terms of a 10-unit (i.e., 10%) *decrease* in the CMI and β_2 in terms of a 10-unit (i.e., 10-day) increase in the number of days since the 100th case in the country.

In a sensitivity analysis, we change the lag between CMI and the outcome to be three weeks rather than two, such that β_1 is the fixed effect of a 1-unit increase in the CMI three weeks prior (x_{1i}) on $log(y_i)$.

An additional sensitivity analysis introduces an interaction term between the fixed effect of CMI and the week (March 16, March 23, March 30, and April 6) of the observation *i*. For the adjusted model, this corresponds to the following equation:

$$log(y_i) = \alpha_{jk[i]} + \beta_1 x_{1i} + \beta_2 x_{2i} + \beta_3 x_{1i} x_{3i} + \beta_4 x_{1i} x_{4i} + \beta_5 x_{1i} x_{5i} + \epsilon_i$$

In this equation, x_{3i}, x_{4i}, x_{5i} are indicator variables for the non-reference weeks (March 23, March 30, April 6), β_1 is the fixed effect of a 1-unit increase in the CMI two weeks prior (x_{1i}) during the reference week (March 16), $\beta_1 + \beta_3$ is the fixed effect of a 1-unit increase in the CMI two weeks prior during the second week (March 23), and so on.

The models for the effective reproduction number outcome are identical to the above except the outcome is y_i, the *i*th (weekly) effective reproduction number, rather than $log(y_i)$, the natural logarithm of the *i*th weekly case ratio.
